# EBV-positive Hodgkin lymphoma is associated with suppression of p21^cip1/waf1 ^and a worse prognosis

**DOI:** 10.1186/1476-4598-9-32

**Published:** 2010-02-09

**Authors:** Ting-Yun Liu, Shang-Ju Wu, Mi-Hsin Huang, Fei-Yun Lo, Mong-Hsun Tsai, Ching-Hwa Tsai, Su-Ming Hsu, Chung-Wu Lin

**Affiliations:** 1 Department of Pathology, National Taiwan University Hospital, National Taiwan University College of Medicine, No.1, Jen-Ai Road, Taipei 100, Taiwan; 2 Internal Medicine, National Taiwan University Hospital, National Taiwan University College of Medicine, No.1, Jen-Ai Road, Taipei 100, Taiwan; 3 Graduate Institute of Biotechnology, College of Bioresources and Agriculture, National Taiwan University, 4F., No.81, Changsing St., Taipei 106, Taiwan; 4 Graduate Institute of Microbiology, National Taiwan University Hospital, National Taiwan University College of Medicine, No.1, Jen-Ai Road, Taipei 100, Taiwan

## Abstract

**Background:**

About 30-50% of Hodgkin lymphomas (HLs) harbor the Epstein-Barr virus (EBV), but the impact of EBV infection on clinical outcomes has been unclear. EBV-encoded small RNAs (*EBER*s) are presented in all EBV-infected cells, but their functions are still less understood.

**Results:**

*EBER1 *was transfected into two HL cell lines, KMH2 and L428, and microarrays were used to screen for *EBER1*-induced changes. We found that *EBER1 *suppressed *p21*^cip1/waf1 ^transcription in HL cell lines. In addition, positive regulators of *p21*^cip1/waf1 ^transcription, such as p53, EGR1, and STAT1, were decreased. Suppression of *p21*^cip1/waf1 ^in the *EBER1*^+ ^HL cell lines was associated with increased resistance to histone deacetylase inhibitors or proteasome inhibitors, drugs known to cause apoptosis by increasing p21^cip1/waf1 ^levels. On biopsy specimens, EBV^+ ^HLs had weaker expression of both p21^cip1/waf1 ^and active caspase 3. Clinically, suppression of p21^cip1/waf1 ^in EBV^+ ^HLs was associated with a worse 2-year disease-free survival rate (45% for EBV^+ ^HLs *vs*. 77% for EBV^- ^HLs, *p *= 0.002).

**Conclusion:**

Although the underlying mechanisms are still relatively unclear, *EBER1 *inhibits *p21*^cip1/waf1 ^transcription and prevents apoptosis through down-regulation of p53, EGR1, and STAT1. The anti-apoptotic activity of *EBER1 *may be important in the rescue of Reed-Sternberg cells from drug-induced apoptosis and in the clinical behaviors of EBV^+ ^HLs.

## Background

In industrialized countries, about 30-50% of Hodgkin lymphomas (HLs) have been associated with the Epstein-Barr virus (EBV), but the impact of EBV infection on the clinical outcomes has been difficult to measure, because most HLs respond well to chemotherapy. In a multicenter retrospective survey, the prognosis was found to be worse for adult EBV^+ ^HLs than for their EBV^- ^counterparts [1]. However, the underlying mechanism is still unknown.

In addition to HL, EBV is also associated with Burkitt's lymphoma, nasopharyngeal carcinoma, and other malignancies [2]. Although EBV can switch its life cycle between a lytic phase and a latent phase [3], the virus exists only in a latent phase in EBV-infected tumor cells. The latent phase is characterized by the variable expression of a limited set of virus-encoded genes, including 6 nuclear antigens (EBNAs 1, 2, 3A, 3B, 3C, & LP), 3 latent membrane proteins (LMPs 1, 2A, 2B), and 2 small homologous RNAs (*EBERs *1& 2). Depending on the expression patterns, the latent phase can be further classified into three types [4]. EBNA1 and *EBERs *are the only EBV-encoded genes common to all latencies. They are probably indispensable for latency maintenance or malignant transformation.

In the latency phase, EBNA1 maintains replication of the episomal form of the virus [5], and it enhances the growth of HL cells [6]. In contrast, the roles of *EBERs *are unclear and controversial. *EBER *probably interacts with both a ribosomal protein L22 and an RNA-dependent protein, PKR [7]. According to one model, PKR may induce apoptosis; *EBERs *antagonize PKR-mediated apoptosis, whereas L22 competes with PKR for *EBERs *binding and abolishes the anti-apoptotic activity of *EBERs*. The anti-apoptotic activity of *EBERs *is consistent with the finding that EBV infection could reduce apoptosis in Burkitt's lymphoma [8,9]. In addition, PKR-independent anti-apoptotic activities of *EBERs *have been reported [10], but the mechanism and clinical significance are still unknown.

To address the mechanism and clinical significance of the anti-apoptotic activity of *EBERs*, we analyzed the *EBER1*-induced changes in HL cell lines using microarrays and found that *EBER1 *suppressed *p21*^cip1/waf1 ^transcription. *p21*^cip1/waf1 ^is also known as the cyclin-dependent kinase inhibitor 1A (CDKN1A), and it normally causes cell cycle arrest at the G1/S phase, and induces or inhibits apoptosis [11-13]. We demonstrated that decreased *p21*^cip1/waf1 ^transcription is associated with increased resistance to drug-induced apoptosis in HL cell lines. Most significantly from a clinical perspective, suppression of *p21*^cip1/waf1 ^and the increased resistance to drug-induced apoptosis are associated with a worse prognosis in cases of EBV^+ ^HLs.

## Methods

### Cell lines

KMH2 and L428, two EBV-negative HL cell lines, were obtained from the German Collection of Microorganisms and Cell Culture (DSMZ, Braunschweig, Germany). Similar to the classical Reed-Sternberg cells in HLs, these cell lines are CD30^+^/CD15^+^/CD3^-^/CD19^-^, and they have rearrangement of the immunoglobulin heavy-chain genes [14]. These cell lines were cultured in RPMI1640 containing 10% fetal bovine serum, 50 μg/mL streptomycin, and 50 U/mL penicillin, at 37°C with 5% CO_2_.

### Construction of plasmids expressing *EBER1 *or antisense-*EBER1 *and selection of stable clones

The plasmid p9362 with an H1 promoter for transcription of small RNAs was used as the expression vector. The plasmid also expressed EGFP, as well as Kanamycin in bacteria or G-418 in eukaryocytes. The 167-nucleotide *EBER1 *or antisense-*EBER1 *was inserted into p9362 for construction of p9362-*EBER1 *or p9362-antisense-*EBER1*. Four cell lines were constructed: KMH2 transfected with p9362-*EBER1 *(KE), L428 transfected with p9362-*EBER1 *(LE), KMH2 transfected with p9362 (K9), L428 transfected with p9362 (L9). One additional control cell line, KMH2 transfected with p9362-antisense-*EBER1 *(K-anti-E), was also established.

Briefly, about 1 × 10^6 ^KMH2 or L428 cells were transfected with 30 μg *EBER1*-expressing plasmid (p9362-*EBER1*), antisense-*EBER1*-expressing plasmid (p9362-antisense-*EBER1*), or control plasmid (p9362) by electroporation with an ECM630 system (BTX, Holliston, MA). Stable clones were selected in RPMI1640 and 10% fetal bovine serum containing 1 mg/mL GENETICIN (Invitrogen, Carlsbad, CA). *EBER1*^+ ^cell lines (KE & LE), an antisense-*EBER1*^+ ^cell line (K-Anti-E), and plasmid-only cell lines (K9 & L9) were established. A purity of greater than 99% of EGFP^+ ^cells was confirmed by flow cytometric analysis and expression of *EBER1 *was confirmed by Northern blotting.

### Northern blotting

Northern blotting was done with dig-labeled probes: 5'-ACAGACACCGTCCTCACCACCCGGGACTTGTACCCGGGACGGGTG-3' for *EBER1 *or 5'-TCTTCTCTGTATCGTTCCAATTTTAGTATATGTGCTGCCG-3' for *U6*.

Briefly, 2.5 μg small RNAs were separated on a 5% denaturing polyacryamide gel and transferred to a Hybond-N membrane (Amersham, Little Chalfont, Bucks, UK). The membrane was hybridized with the *EBER1 *or *U6 *probe at a concentration of 50 ng/mL in a buffer containing 50% formamide at 52°C for 16 hours. The membrane was then washed twice with 2 × SSC in 0.1% SDS at 25°C for 5 min, and twice with 0.2 × SSC in 0.1%SDS at 68°C for 10 min. Anti-digoxigenin-AP and CSPD (Roche, Mannheim, Germany) were used for development of chemiluminescence.

### Microarray

The Affymetrix chip, Human Genome U133 plus 2.0, was used to obtain genome-wide transcriptional profiles of the four stable cell lines (K9, KE, L9, and LE). First-strand cDNAs were synthesized from 10 μg of total RNAs with a T7-promoter-oligo (dT) primer. After second-strand synthesis, biotin-labeled cRNAs were transcribed from the T7 promoter. The cRNAs were fragmented into sizes ranging from 35 to 200 nucleotides, labeled with streptavidin-PE, mixed with control RNAs (bioB, bioC, bioD, and cre), and hybridized with the glass slides according to the GeneChip Expression Analysis Technical Manual from Affymetrix. The arrays were scanned with GenePix 4000B (Molecular Devices, Sunnyvale, CA, USA), and the data were extracted with Affymetrix Microarray Suite (MAS) software and submitted to Gene Expression Ominbus at http://www.ncbi.nlm.nih.gov/geo/ with the accession number GSE12427.

Local normalization of the extracted raw data was done online at http://pevsnerlab.kennedykrieger.org/snomadinput.html[15]. In this method, the significance of the difference between the *EBER1*^+ ^cell lines and the control cell lines was designated by the z-score. For example, the *p21*^cip1/waf1 ^transcripts had a 2-fold decrease from 2260 in K9 to 1164 in KE, a mean level of 1712, and a z-score, Z_K_, of - 2.5. The z-score meant that the 2-fold decrease for *p21*^cip1/waf1 ^was located at -2.5 standard deviations, when normalized with respect to the changes of genes with a similar mean level of expression. Each gene thus had a Z_K _for KE *vs*. K9, and a second Z_L _for LE *vs*. L9. The changes for the gene were concordant if both z-scores were positive or both z-scores were negative.

### RT-PCR for p21^cip1/waf1 ^splicing variants

Eight splicing variants of *p21*^cip1/waf1 ^have been reported: variant 1, variant 2, Alt-a, Alt-a', Alt-b, Alt-c, B, and C [16,17]. A universal RT primer, 5'-RS-CATTAGCGCATCACAGTCGC-3' (5506-5487), was used for converting mRNAs of all splicing variants into cDNAs. This RT primer consists of a *p21*^cip1/waf1 ^binding sequence tagged with a random sequence (RS): 5'-*GTATACTGCAGGGTCTGATAC*-3'. A fluorescent universal reverse PCR primer, 5'-FAM-ATAGGTATACTGCAGGGTCTGATAC-3', and a forward PCR primer specific for each variant were then used to amplify the cDNAs. The specific forward primers were: 5'-CTGCCGAAGTCAGTTCCTTG-3' (variant 1, 18~37), 5'-ACTCAGAGGAGGTGAGAGAG-3' (variant 2, 79~98), 5'-GGTGGCTATTTTGTCCTTGG-3' (Alt-a, a' and b, - 835~-814), 5'-GGAGGCAAAAGTCCTGTGTT-3' (Alt-c, - 2219~-2197), 5'-AAGGAGGAGAGAGACCCT CT-3' (B, 5266~5286), and 5'-CTAGAAAATCCAGTTGCTG-3' (C, 3954~3972). RT-PCR for *β2M *was used as an internal control. A reverse primer, 5'-RS-CAGAATTTGGAATTCATCCAA-3', and a forward primer, 5'-CTTTGTCACAGCCCAAGATAG-3', were used. The numbers in parentheses are the locations of the primers, with respect to the genomic position of the *p21*^cip1/waf1^, with the transcription start site of variant 1 being +1.

The PCR products were separated by high-resolution capillary electrophoresis and quantified by fluorescence. The size of the PCR products in base pairs were: 216 for variant 1, 296 for variant 2, 179 for alt-a, 225 for alt-a', 191 for alt-b, 349 for alt-c, 208 for B, and 213 for C. The 3 variants (alt-a, a' and b) from the same PCR had different sizes due to alternative splicing.

### Real-time RT-PCR for p21^cip1/waf1^

Taqman^® ^Gene Expression Assays (Applied Biosystems, Foster City, CA) were used for real-time RT-PCR of *p21*^cip1/waf1 ^(HS_0001121172_m1) and *actin *(HS_00357333_g1). Total RNAs were extracted from K9, KE, L9, and LE cells. The RNAs were reverse transcribed into cDNAs with random hexamers. After an initial 10-min denaturation step at 95°C, 45 cycles of PCR were performed with denaturation at 95°C for 15 sec and annealing at 60°C for 1 min on the Stepone™ Real-Time PCR system (Applied Biosystems, Foster City, CA). The threshold cycle of *p21*^cip1/waf1 ^minus that of *actin *was calculated.

### Western blotting for p21^cip1/waf1^, EGR1, STAT1, p53, SirT5, GAPDH, cyclin-dependent kinases (CDKs) and cyclins

Western blotting was performed with the following antibodies: p21^cip1/waf1 ^(clone CP74, Lab Vision, Fremont, CA), SirT5 (rabbit polyclonal, Abcam, Cambridge, UK), STAT1 (clone 42H3, Cell signaling, Danvers, MA), EGR1, p53, CDK6, and GAPDH (clone 588, clone DO-1, clone C-21, and clone FL-335, SANTA CRUZ, Santa Cruz, CA), CDK1, CDK2, CDK4, and cyclin B1 (clone POH-1, clone Poly6332, clone Poly6333, and clone Poly6334, Biolegend, San Diego, CA), cyclin A (clone 25/CyclinA, BD Pharmingen, Franklin Lakes, NJ), cyclin D2, and cyclin E (clone DCS-3.1, and clone HE12, Abcam, Cambridge, UK).

### Cell cycle analysis

Cells were cultured in RPMI1640 medium plus 10% fetal bovine serum for 24 hours. The cells were fixed with 75% ethanol at -20°C overnight and were stained in 50 μg/mL propidium iodide (Sigma, Saint Louis, MO, USA), 0.05% Triton X-100, 0.1 μg/μL RNase A, and 1× PBS at 37°C for 30 min in the dark. The stained cells were washed with 3 mL PBS and suspended in 500 μL PBS for flow-cytometric analysis.

### Apoptosis induced by TSA & MG115 measured by flow cytometry for Annexin V and propidium iodide

TSA (Trichostatin A, a histone deacetylase inhibitor from Sigma, Saint Louis, MO, USA) and MG115 (a proteasome inhibitor from Calbiochem, San Diego, CA) were used to induce apoptosis through up-regulation of p21^cip1/waf1^. About 1 × 10^5 ^KE, K9, LE, or L9 cells were grown in 1 mL of medium containing by 0.5 μM TSA or by 0.4 μM MG115. After 2 days, the cells were harvested, washed twice with 1× PBS, and suspended in 100 μL 1× binding buffer containing 10 mM Hepes at pH 7.4, 140 mM NaCl, and 2.5 mM CaCl_2_. The cells in 100 μL binding buffer were mixed with 5 μL APC-conjugated Annexin V (BD Pharmingen, Franklin Lakes, NJ) and 5 μL of 50 μg/mL propidium iodide at 25°C for 15 min. The stained cells were diluted with 400 μL of 1× binding buffer and analyzed by flow cytometry within 1 hour.

### Induction of p21^cip/waf1 ^by TSA or MG115 measured by ELISA

K9, KE, L9, LE cells were treated with 0.5 μM TSA or 0.4 μM MG115 for 1 day. Untreated cells were used as controls. The amounts of p21^cip/waf1 ^were measured with an ELISA kit (Total p21^cip/waf1 ^Sandwich ELISA Kit, Cell Signaling, Danvers, MA). The ratio of p21^cip/waf1 ^in the treated cells to that in the untreated cells was calculated.

Briefly, after cell lysis and protein extraction, 25 μg proteins were loaded onto p21^cip/waf1 ^antibody-coated microwells at 37°C for 2 hours. A detection antibody for p21^cip/waf1^, an HRP-linked secondary antibody, and the TMB substrate were applied sequentially. The absorbance at 450 nm was measured, and the background absorbance was subtracted out. The ratios of the absorbances of treated cells to those of untreated cells were calculated.

### Cell growth curve

For growth curve, 1 × 10^4 ^K9, KE, L9, or LE cells were grown in 0.5 mL medium containing 0 to 0.5 μM TSA or 0 to 0.4 μM MG115 for up to 2 days. The cells were stained with 0.4% trypan blue and counted by hemocytometer.

### Tissue samples

Biopsy specimens of 94 HLs with sufficient tissues and clinical data for further investigations were retrieved from the lymphoma database at the Department of Pathology of the National Taiwan University Hospital. The study was approved by the ethics committee of the National Taiwan University Hospital.

### Immunohistochemistry for p21^cip1/waf1 ^and in situ hybridization for *EBER1*

Immunoperoxidase stain for p21^cip1/waf1^, active caspase 3, and Ki-67 were performed on sections of formalin-fixed, paraffin-embedded HL cell blocks and tissue samples with the antibodies to p21^cip1/waf1 ^(clone EA10, Calbiochem, Darmstadt, Germany), active caspase 3 (C92-605, BD Pharmingen, Franklin Lakes, NJ), and Ki67 (MIB-1, DAKO, Glostrup, Denmark). For each case, 50 Reed-Sternberg cells were examined, and the percentages of positive cells were recorded.

In situ hybridization for *EBER1 *was done on formalin-fixed, paraffin-embedded tissue sections with a dig-labeled probe, 5'-ACAGACACCGTCCTCACCACCCGGGACTTGTACCCGGGACGGGTG-3'. The sections were detected with immunoalkaline phosphatase and developed with BCIP/NBT.

### Statistical analysis

The clinical data were extracted from the medical records. Two-sample comparisons were done with the Fischer's test for categorical data and the Mann-Whitney test for continuous data. 2-year overall survival (OS) rate and disease-free survival (DFS) rate analyses were done with the Kaplan-Meier method.

## Results

### *EBER1 *suppressed transcription of p21^cip1/waf1^

**Figure 1:**
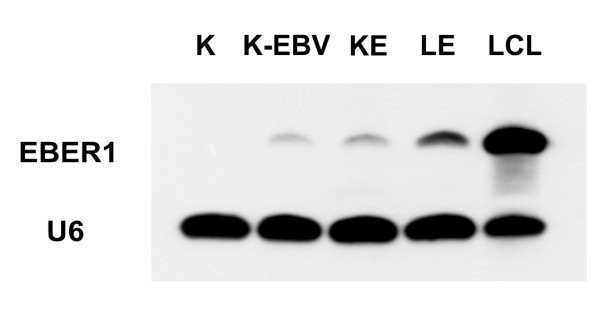
**Northern blotting demonstrated *EBER1 *expression in KE and LE**. The expression of *EBER1 *was measured by Northern blotting in the KMH2 cell line (K), an EBV-infected KMH2 cell line (K-EBV), KE, LE, and an EBV-infected lymphoblastoid cell line (LCL). The expression of *EBER1 *in KE was similar to that of K-EBV. The expression of *EBER1 *in LE was higher than that of KE, but lower than that of LCL.

*EBER1*-expressing plasmids (p9362-*EBER*) or control plasmids (p9362) were transfected into KMH2 and L428 cells. Four stable cell lines were established: *EBER1*^+ ^KMH2 (KE), *EBER1*^+^L428 (LE), p9362-KMH2 (K9), and p9362-L428 (L9). As shown in Fig 1, the expression of *EBER1 *in KE was similar to that of an EBV-infected KMH2 cell line (K-EBV). The expression of *EBER1 *in LE was higher than that of KE but lower than that of an EBV-infected lymphoblastoid cell line (LCL).

Microarrays were used to screen for *EBER1*-induced changes, and the changes were measured by the z-score [15] A positive score was given if an increase was induced by *EBER1*, and a negative score was given if a decrease was induced by *EBER1*. A gene was therefore given a z-score, Z_K_, for the changes between KE & K9, and a second z-score, Z_L_, for the changes between LE & L9.

Because true physiologic actions of *EBER1 *should be induced in both KE and LE cell lines, Z_K _and Z_L _should be concordantly increased or decreased. For facilitating the identification of such concordant changes, the z-scores, Z_K _& Z_L_, were multiplied. The genes were then listed according to the values of Z_K _*Z_L_. The top 10 genes with the largest concordant decrease or increase are listed in Table 1. Among these top-ranking genes in Table 1, *EGR1 *and *p21*^cip1/waf1 ^appeared to be functionally related, because EGR1 can activate *p21*^cip1/waf1 ^transcription [18,19].

**Table 1:** Genes concordantly down- or up-regulated by *EBER1 *in KMH2 & L428

Unigene	Gene Title	K9	KE	L9	LE	Z_K_	Z_L_	Z_K_*Z_L_
**Top 10 down-regulated genes**								

**Hs.326035**	Early growth response 1	379	110	3992	437	-3.8	-6.1	23.2
**Hs.370771**	Cyclin-dependent kinase inhibitor 1A (p21, Cip1)	2260	1164	613	18	-2.5	-7.6	19.4
**Hs.440366**	Similar to sirtuin 5 isoform 2; sir2-like 5	340	93	1416	201	-3.9	-4.7	18.5
**Hs.56382**	Hexamthylene bis-acetamide inducible 2	208	54	316	24	-3.4	-5.3	18.1
**Hs.3210**	Renin	483	21	93	20	-7.8	-2.2	17.0
**Hs.143757**	Similar to zinc finger protein 111	450	123	258	42	-4.1	-3.7	15.1
**Hs.199877**	Copine IV	454	154	2186	449	-3.6	-4.1	14.7
**Hs.501778**	Tripartite motif-containing 22	1200	606	554	47	-2.7	-5.4	14.5
**Hs.349110**	Hepatocyte growth factor-like	184	50	317	50	-3.2	-3.8	12.4
**Hs.129867**	Calcium and integrin binding family member 2	150	52	213	15	-2.6	-4.8	12.2

**Top 10 up-regulated genes**								

**Hs.292788**	Homo sapiens, clone IMAGE:5189562, mRNA	10	167	22	180	3.7	4.9	18.1
**Hs.147434**	TRAF3 interacting protein 3	175	605	446	3676	2.8	6.4	17.9
**Hs.550344**	CDNA FLJ32691 fis, clone TESTI2000221	25	185	66	250	3.9	3.9	15.4
**Hs.163914**	Hypothetical protein DKFZp313G1735	14	138	15	160	3.2	4.6	14.8
**Hs.154510**	Chr 2 open reading frame 4///Carbonyl reductase 3	15	97	11	244	2.3	6.2	14.2
**Hs.247831**	Myosin light chain 2, precursor lymphocyte-specific	77	384	82	308	3.5	4.0	14.0
**Hs.268939**	Matrin 3	254	698	18	219	2.3	5.8	13.1
**Hs.515011**	SMAD specific E3 ubiquitin protein ligase 2	67	232	114	580	2.7	4.8	13.0
**Hs.512709**	Troponin I type 3 (cardiac)	34	206	31	114	3.9	3.1	12.1
**Hs.530863**	CASK interacting protein 1	19	126	35	162	2.9	4.0	11.8

From left to right, the columns show the UniGene number, the gene title, the gene symbol, the levels of the transcripts in K9, KE, L9, and LE, the z-score (Z_K_) for the comparison between K9 & KE, the z-score (Z_L_) between L9 & LE, and the product of Z_K _*Z_L_.

### Transcription factors of p21^cip1/waf1^, such as *EGR1 *&*STAT1*, were also decreased by *EBER1*

**Figure 2:**
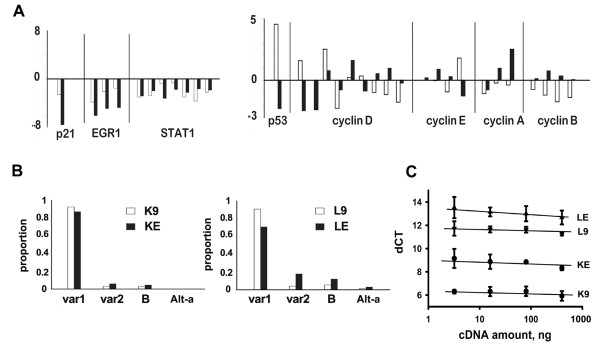
***EBER1 *decreased *p21*^cip1/waf1 ^transcription in HL cell lines**. 2A: Transcript profiles of *p21*^cip1/waf1^-related genes. The z-scores, Z_K _in white and Z_L _in black, were plotted for the following genes: 1) *p21*^cip1/waf1 ^& its regulators: *EGR1 *and *STAT1*, 2) cell cycle-related genes: *p53 *and *cyclins*. Note that the Affymetrix chip used multiple sets of probes to monitor a single gene, but only *EGR1 *and *STAT1 *showed consistent decreases across all probe sets. 2B: Eight splicing variants of *p21*^cip1/waf1 ^have been reported: variant 1, variant 2, Alternative-a (Alt-a), Alternative-a' (Alt-a'), Alternative-b (Alt-b), Alternative-c (Alt-c), B, and C. *EBER1 *did not change the splicing patterns, as RT-PCR showed that variant 1 was always the dominant form for K9, KE, L9, and LE. X-axis: *p21 *variants; Y-axis: proportions of the variants. Only the major variants, variant 1, variant 2, B, and Alt-a were shown. The others variants were all less than 1%. 2C: Representative real-time RT-PCRs for *p21*^cip1/waf1 ^were shown. X-axis: cDNA amount; Y-axis: dCT (the threshold cycle of *p21*^cip1/waf1 ^minus that of *actin)*. Triplicate measurements were taken at each one of 4 cDNA concentrations. The dCT (mean +/- SD) was 6.2 +/- 0.2 for K9, 8.7 +/- 0.4 for KE, 11.5 +/- 0.2 for L9, and 13.0 +/- 0.4 for LE.

In Fig 2A, the transcripts of *p21*^cip1/waf1^-related genes were analyzed in details, such as *EGR1*, *STAT1 *[20], *p53 *[21,22], and *cyclins*. Significantly, *EGR1 *and *STAT1 *were decreased by *EBER1 *across all probe sets that were used in the Affymetrix chip to monitor a single gene. In contrast, *p53*, a physiologic regulator of *p21*^cip1/waf1^, and *cyclins*, the physiologic targets of *p21*^cip1/waf1^, did not have consistent changes. These preliminary observations indicated that *EBER1*-induced *p21*^cip1/waf1 ^suppression was related to *EGR1 *and *STAT1*, but not necessarily to *p53*, and did not lead to consistent changes in *cyclins*.

### *EBER1 *suppressed *p21*^cip1/waf1 ^transcription

To confirm the microarray data and to exclude the possibility of *EBER1*-induced changes in alternative splicing patterns, we used RT-PCR for a semi-quantitative assessment of all 8 splicing variants of *p21*^cip1/waf1^. Variant 1 was always the dominant form (Fig 2B). Real-time RT-PCR (Fig 2C) further showed the dCT (mean +/- SD) was 6.2 +/- 0.2 for K9, 8.7 +/- 0.4 for KE, 11.5 +/- 0.2 for L9, and 13.0 +/- 0.4 for LE. Pairwise comparisons between the cell lines were all significant at *p *< 0.001 by the t-test. Thus K9 had more *p21*^cip1/waf1 ^transcripts than L9, and the presence of *EBER1 *decreased *p21*^cip1/waf1 ^in both cell lines. The relative levels, K9>KE>L9>LE, were consistent with the results from microarrays. Together, these data showed that *EBER1 *suppressed *p21*^cip1/waf1 ^transcription without altering alternative splicing.

### *EBER1 *suppressed the expressions of *p21*^cip1/waf1^, EGR1, STAT1, & p53

**Figure 3:**
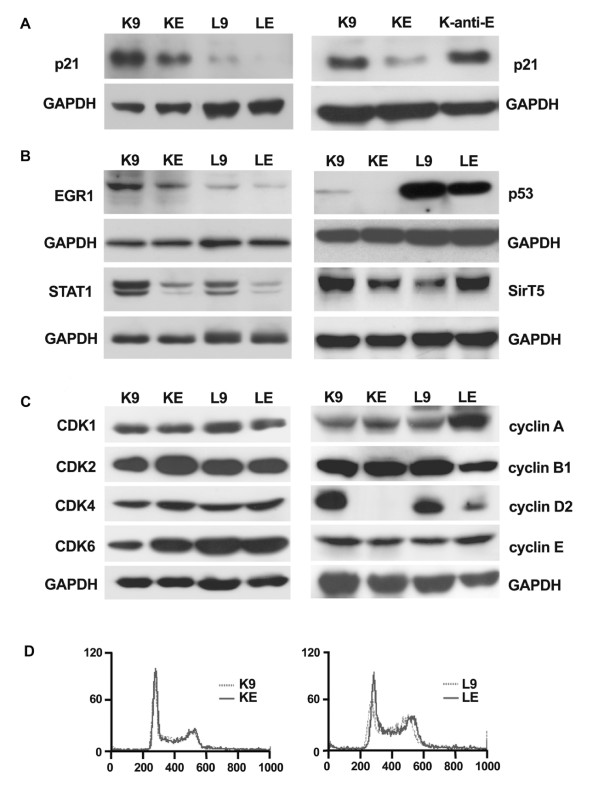
***EBER1 *suppressed p21^cip1/waf1 ^without changes in cell cycle distributions**. 3A: *EBER1 *suppressed p21^cip1/waf1^. Left panel: *EBER1 *decreased p21^cip1/waf1 ^in KE and LE, although overloading was necessary to show the much weaker levels of p21^cip1/waf1 ^in L9 & LE. Right panel: *EBER1 *but not antisense *EBER1 *suppressed p21^cip1/waf1^. 3B: *EBER1 *suppressed EGR1, STAT1, and p53, but had no consistent effects on SirT5. The doublets of STAT1 were due to isoforms. 3C: Western blotting showed decreased cyclin D2 in KE & LE, but no changes in the levels of CDKs or other cyclins. 3D: Cell cycle distributions of K9 (dash) and KE (solid) on the left, L9 (dash) and LE (solid) on the right. There were no detectable differences in the cell cycle distributions between KE & K9, or LE & L9. The Y-axis is the number of cells. The X-axis is the intensity of propidium iodide (PI) on a linear scale from zero to 1000. The first peak at around 250 had diploid DNA content (2N) and was the peak of the G0/G1 phase. The second peak at around 500 had quadruploid DNA content (4N) and was the peak of the G2/M phase. Between these 2 peaks, cells were in S phase.

Western blotting was used for confirming the microarray data at the levels of protein expression. In Fig 3A, *EBER1 *decreased p21^cip1/waf1 ^in both KE and LE cell lines. In a KMH2 cell line expressing antisense *EBER1*, the expression of p21^cip1/waf1 ^protein was not suppressed. Thus the suppression on p21^cip1/waf1 ^was not a nonspecific effect of RNA, but required the specific sequence of *EBER1*.

In Fig 3B, the positive regulators of *p21*^cip1/waf1 ^transcription, such as EGR1 & STAT1, were decreased by *EBER1*. Unexpected from the array data, p53, another positive regulator of *p21*^cip1/waf1 ^transcription, was also decreased in both KE & LE cell lines.

Because *p21*^cip1/waf1 ^transcription was reported to be up-regulated by histone acetylation [23,24], Western blotting for the histone deacetylase, SirT5, was performed. SirT5 was decreased in the KE cell line, but increased in the LE cell line. Further experiments are thus necessary for clarifying the role of SirT5.

### *EBER1*-induced *p21*^cip1/waf1 ^suppression was associated with decreased cyclin D2, but did not change the cell cycle distributions

Because p21^cip1/waf1 ^may arrest the cell cycle at the G1/S transition, we investigated the effect of suppressed p21^cip1/waf1 ^on the cell cycle. We found decreased cyclin D2 in KE & LE, but not other CDKs & cyclins (Fig 3C). Cyclin D2 normally peaks at the late G1 phase and promotes the G1/S transition. The simultaneous decrease of cyclin D2 and p21^cip1/waf1 ^may have opposite effects on the G1/S transition, resulting in no net changes in the cell cycle distributions (Fig 3D).

### *EBER1 *conferred resistance to apoptosis induced by TSA & MG115

The histone deacetylase inhibitor TSA increases *p21*^cip1/waf1 ^transcription [24], and the proteasome inhibitor MG115 increases p21^cip1/waf1 ^by abolishing protein degradation [25]. Both have been used extensively in treatment of lymphoma, because of their ability to induce apoptosis [26,27].

**Figure 4:**
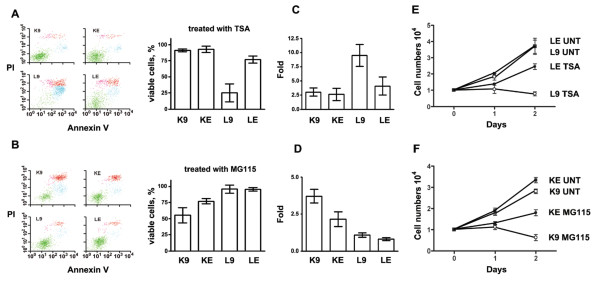
***EBER1 *conferred resistance to apoptosis induced by TSA & MG115**. 4A & 4B: Left panel: Apoptosis induced by 0.5 μM TSA (4A) or 0.4 μM MG115 (4B) for 2 days was measured by flow cytometry. The X-axis is Annexin-V (AV) and the Y-axis is propidium iodide (PI). Both the X and Y-axes are in log scale from 10^0 ^to 10^4^. Green: AV^-^/PI^-^ viable cells. Blue: AV^+^/PI^dim ^apoptotic cells. Red: AV^+^/PI^bright ^dead cells. Right panel: The percentages of viable cells after treatments were measured. (Y-axis: percentage of viable cells; X-axis: cell lines). Note that L9 was the most sensitive cell line after TSA treatment (4A), and K9 was the most sensitive cell line after MG115 treatment (4B). 4C & 4D: Induction of p21^cip1/waf1 ^by 0.5 μM TSA (4C) or by 0.4 μM MG115 (4D) was measured with ELISA. (Y-axis: p21^cip1/waf1 ^of treated cell lines divided by that of untreated cell lines; X-axis: cell lines). Note that L9 showed the highest induction after TSA treatment and K9 showed the highest induction after MG115 treatment. 4E & 4F: After treatment with 0.5 μM TSA, L9 cells grew slower than LE cells(4E), and after treatment with 0.4 μM MG115, K9 cells grew slower than KE cells (4F). (Y-axis: cell numbers in 10^4^; X-axis: duration of culture in days; UNT: untreated).

The susceptibility of K9, KE, L9, and LE to drug-induced apoptosis was tested. Triplicate measurements by flow cytometry showed that the percentages of viable cells (mean +/- SD) after TSA treatment (Fig 4A) were 91.1 +/- 2.0 for K9, 93.0 +/- 4.9 for KE, 25.2 +/- 13.6 for L9, and 76.9 +/- 5.3 for LE. L9 and LE had less viable cells than K9 and KE. Significantly, L9 was even less viable than LE (*p *= 0.04, paired t-test). Similarly, the percentages of viable cells (mean +/- SD) after MG115 treatment (Fig 4B) were 55.1 +/- 11.7 for K9, 76.9 +/- 7.3 for KE, 95.9 +/- 6.2 for L9, and 95.3 +/- 2.6 for LE. K9 and KE had less viable cells than L9 and LE. Significantly, K9 was even less viable than KE (*p *= 0.03, paired t-test). Thus, L9 was more sensitive to TSA than LE (Fig 4A), and K9 was more sensitive to MG115 than KE (Fig 4B). The differential sensitivities implied that intrinsic properties of the cell lines could have caused L9/LE to be more sensitive to TSA than K9/KE, and K9/KE to be more sensitive to MG115 than L9/LE.

ELISA was used to measure the induction of p21^cip1/waf1 ^after drug treatments. The increase (mean +/- SD, triplicate measurements) after TSA treatment (Fig 4C) was 3.0 +/- 0.7 fold in K9 cells, 2.6 +/- 1.0 fold in KE cells, 9.5 +/- 1.9 fold in L9 cells, and 4.0 +/- 1.6 fold in LE cells. L9 and LE had more induction of p21^cip1/waf1 ^than K9 and KE, and significantly L9 showed higher induction of p21^cip1/waf1 ^than LE (*p *= 0.02, paired t-test). Similarly, the increase (mean +/- SD, triplicate measurements) after MG115 treatment (Fig 4D) was 3.7 +/- 0.4 fold in K9 cells, 2.1 +/- 0.5 fold in KE cells, 1.0 +/- 0.1 fold in L9 cells, and 0.8 +/- 0.1 fold in LE cells. K9 and KE had more induction of p21^cip1/waf1 ^than L9 and LE, and significantly K9 showed higher induction of p21^cip1/waf1^than KE (*p *= 0.01, paired t-test). Thus, consistent with the data on apoptosis, L9 cells had the most significant increase of p21^cip1/waf1 ^under TSA treatment (Fig 4C) and K9 cells had the most significant increase of p21^cip1/waf1 ^under MG115 treatment (Fig 4D).

The effects of TSA and MG115 on cell growth were measured. Based on 4 repeats, the cell numbers at 48 hours (mean +/- SD in 10^4^) after TSA treatment (Fig 4E) were 3.6 +/- 0.4 for untreated LE, 3.7 +/- 0.5 for untreated L9, 2.4 +/- 0.1 for treated LE, and 0.7 +/- 0.1 for treated L9. Treated L9 cells grew slower than the others (*p *< 0.001, one-way ANOVA). Similarly, the cell numbers at 48 hours (mean +/- SD in 10^4^) after MG115 treatment (Fig 4F) were 3.3 +/- 0.1 for untreated KE, 2.8 +/- 0.1 for untreated K9, 1.8 +/- 0.1 for treated KE, and 0.6 +/- 0.1 for treated K9. Treated K9 cells grew slower than the others (*p *< 0.001, one-way ANOVA). Thus, L9 grew slower than LE cells when treated with TSA (Fig 4E), whereas K9 grew slower than KE cells when treated with MG115 (Fig 4F).

Taken together, these data were consistent with the hypothesis that *EBER1 *suppressed *p21*^cip1/waf1 ^transcription and conferred resistance to drug-induced apoptosis in these model systems.

### EBV^+ ^Hodgkin lymphoma is associated with suppression of p21^cip1/waf1 ^and a worse prognosis

**Figure 5:**
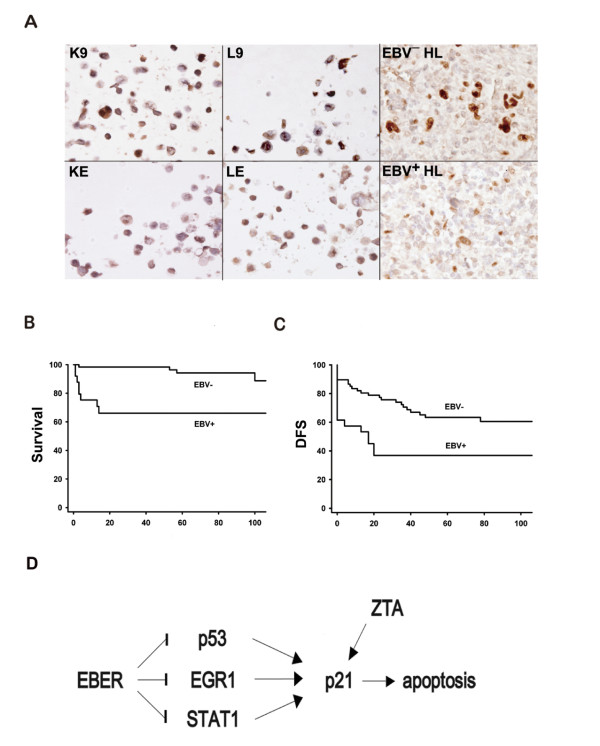
**EBV Hodgkin lymphomas is associated with suppression of p21^cip1/waf1 ^and a worse prognosis**. 5A: Immunohistochemistry of p21^cip1/waf1^. Upper panel from left to right: K9, L9, and an EBV^—^ HL were positive for p21^cip1/waf1^. Lower panel from left to right: KE, LE, and an EBV^+ ^HL were negative for p21^cip1/waf1^. Immunoperoxidase stains on formalin-fixed, paraffin-embedded cell blocks or tissue sections. 5B: The EBV^+ ^group also had a worse 2-year OS rate than did the EBV^— ^group (68% *vs*. 98%, *p *< 0.001 by logrank test). 5C: The EBV^+ ^group had a worse 2-year DFS rate than did the EBV^— ^group (45% *vs*. 77%, *p *= 0.002 by logrank test). 5D: *EBER1 *inhibits apoptosis and maintains latency through *p21*^cip1/waf1 ^suppression (Arrow: increase; Bar: decrease). Our data are consistent with the model that *EBER1 *could inhibit *p21*^cip1/waf1 ^transcription through EGR1, STAT1, or p53. The inhibition of p21^cip1/waf1 ^is associated with resistance to drug-induced apoptosis. Because p21^cip1/waf1 ^is necessary for lytic induction by EBV-encoded ZTA [36,37], *EBER1 *may be critical for the maintenance of the latency phase as well. Since the binding sites on the *p21*^cip1/waf1 ^promoter for EGR1 [18,19], STAT1 [20], and p53 [21,22] are already known, further studies are necessary to reveal how *EBER1 *suppresses p53, EGR1, and STAT1, and whether or not post-transcriptional regulations of *p21*^cip1/waf1 ^are also involved.

Ninety-four HLs, including 68 EBV^- ^and 26 EBV^+ ^cases, were used for the assessment of the clinical significance of p21^cip1/waf1 ^suppression (Fig 5 and Table 2). Immunohistochemical stains were performed for p21^cip1/waf1 ^(Fig 5A), active caspase 3 as an apoptotic marker [28], and Ki67 as a proliferation marker. The percentages of Reed-Sternberg cells that were positive for p21^cip1/waf1 ^were determined, and the median values for the EBV^+ ^and the EBV^- ^groups were listed in Table 2. Compared with the EBV^- ^group, the EBV^+ ^group was slightly more likely to present at a later stage and a higher LDH level (*p *= 0.09 and 0.08, respectively). The EBV^+ ^group expressed significantly less p21^cip1/waf1 ^(44% *vs*. 76%, *p *< 0.001) and active caspase 3 (4% *vs*. 22%, *p *< 0.001), but had a similar amount of Ki67 (52% *vs*. 54%, *p *= 0.80).

**Table 2:** Suppression of p21^cip1/waf1 ^in EBV^+ ^Hodgkin lymphoma is associated with a worse prognosis

	EBV^-^	EBV^+^	*p*
**Clinical Manifestation**			
**n**	68	26	
**age**	34	44	0.18
**sex**	33:35	19:7	0.04
**stage, I&II**	72%	50%	0.09
**LDH**	459	544	0.08
**ABVD**	70%	58%	0.32
**Primary refractory**	9% (6/68)	38% (10/26)	0.002
**2-year OS**	98%	68%	<0.001
**2-year DFS**	77%	45%	0.002
**Biological markers**			
**p21^cip1/waf1^**	76%	44%	<0.001
**active caspase 3**	22%	4%	<0.001
**Ki67**	54%	52%	0.80

From top to bottom, the rows which show clinical manifestation are: number of cases, median age in years, sex ratio in male: female, percentage of cases in stages I & II, median LDH (lactate dehydrogenase, normal at 230-460 U/L), percentage of cases initially treated with ABVD (Adriamycin, Bleomycin, Vinblastine, & Dacarbazine), percentage of primary refractory cases, estimated 2-year overall survival rate, and 2-year disease-free survival rate. The rows which show biological markers are: median percentage of Reed-Sternberg cells that were positive for p21^cip1/waf1^, active caspase 3, and Ki67.

The 2-year OS rate (98% *vs*. 68%, *p *< 0.001, Fig 5B) and 2-year DFS rate (77% *vs*. 45%, *p *= 0.002, Fig 5C) were both better in the EBV^- ^group than in the EBV^+ ^group. In our series, there were 6 primary refractory cases in the EBV^- ^group and 10 primary refractory cases in the EBV^+ ^group (9% *vs*. 38%, *p *= 0.002). The much higher frequency of primary refractory cases in the EBV^+ ^group was the main reason for the worse prognosis.

Although EBV^+ ^and EBV^- ^HLs differed in several parameters and the clinical observations did not establish a casual link, these findings were consistent with the hypothesis that suppression of p21^cip1/waf1 ^allowed the tumor cells in EBV^+ ^HLs to escape spontaneous apoptosis or to resist drug-induced apoptosis, resulting in a more aggressive clinical behavior.

### An anti-apoptotic model for *EBER1*-induced *p21*^cip1/waf1 ^suppression

To integrate our data, we present an anti-apoptotic model (Fig 5D), in which *EBER1 *suppresses *p21*^cip1/waf1 ^transcription indirectly through EGR1, and STAT1. In addition, *EBER1 *might suppress *p21*^cip1/waf1 ^through the wild-type p53 in KE, but not the mutant-type p53 in LE [29,30]. Since the binding sites on the *p21*^cip1/waf1 ^promoter for EGR1 [18,19] and STAT1 [20] and p53 [21,22] are already known, further studies are necessary to reveal how *EBER1 *suppresses p53, EGR1, and STAT1, and whether or not post-transcriptional regulations of *p21*^cip1/waf1 ^are also involved.

## Discussion

We found that *EBER1 *suppresses *p21*^cip1/waf1 ^transcription and inhibits drug-induced apoptosis, but does not change the cyclins [31] except for cyclin D2. The anti-apoptotic activity of *EBER1 *could be critical in the rescue of HL cells from apoptosis. These cells have crippled immunoglobulin genes and should have undergone apoptosis in the germinal center [32]. The increased resistance to drug-induced apoptosis offers a possible explanation for the worse clinical behavior of EBV^+ ^HLs [1].

Although most HLs respond to chemotherapy, about 25% of HLs are refractory or relapse after an initial response [1]. For these cases, it is important to identify prognostic factors, such as the sites and extent of relapse, and to adjust treatment accordingly. Apart from these obvious clinical predictors, biological predictors, such as suppression of p21^cip1/waf1^, should be useful. In fact, the clinical usefulness of p21^cip1/waf1 ^as a prognostic factor has been reported repeatedly for other tumors [33], and our data suggested possible use of p21^cip1/waf1 ^as a prognostic factor in EBV^+ ^HL.

In our study, we have used TSA, a histone deacetylase inhibitor, and MG115, a proteasome inhibitor, to test the effect of *EBER1 *on drug-induced apoptosis in HL cell lines, because similar drugs are currently being evaluated for clinical usages. Bortezomib is a proteasome inhibitor found to cause cell cycle arrest and to induce apoptosis in HL cell lines. However, in a pilot study, the drug demonstrated minimal activity in relapsed and refractory HLs [26]. Similar to the conclusion of this pilot study, our data imply that treatment with proteasome inhibitors in EBV^+ ^refractory/relapsed HLs is likely to be ineffective. Vorinostat is a histone deacetylase inhibitor, which was found to suppress p21^cip1/waf1^, cause cell cycle arrest, and induce apoptosis in HL cell lines [27]. Because we have compared only *EBER1*^+ ^and EBER1^- ^HL cell lines, whereas the Reed-Sternberg cells in EBV^+ ^HLs also express EBNA1 and LMPs, it would be interesting to see whether these drugs are effective in EBV^+ ^HLs in future clinical trials.

From the perspective of tumor biology, there are 3 subtypes of latency in EBV-infected tumor cells. EBV^+ ^HL is typical of type II latency, and only a limited set of virus-associated genes is expressed. These genes include *EBNA1, LMP1 *&*LMP2*, and *EBER*s. EBNA1 keeps the viral genome in an episomal form, LMP1 transmits CD40-like signals to compensate for the lack of B-cell receptors [34], and LMP2A is a B-cell receptor mimic that is essential for survival [35]. Because *EBER1 *may suppress *p21*^cip1/waf1 ^transcription, and p21^cip1/waf1 ^is necessary for lytic induction by EBV-encoded ZTA [36,37], *EBER1 *may be critical for the maintenance of the latency phase.

To integrate the data on apoptosis, drug-resistance, and maintenance of the latency phase, we have presented a model of *EBER1*-induced p21^cip1/waf1 ^suppression through EGR1, and STAT1 (Fig 5D). Other genes in Table 1 might be related to this model too. Sir-2-like 5 (SirT5) is a histone deacetylase that could suppress *p21*^cip1/waf1 ^transcription [23,24]. HEXIM2, a double-stranded RNA-binding protein [38], and MATR3, which processes double-stranded RNAs [39], could have interacted with *EBER1*. Finally, TRIM22 is important for antiviral defense [40].

In addition to HL, Burkitt's lymphoma and post-transplantational lymphoproliferative disorder are also EBV-associated B-cell lymphomas that share a similar pathogenetic mechanism, in which EBV infection is important in the immortalization and transformation of B cells. In experimental conditions, EBV infection of primary B cells leads to p21^cip/waf1^suppression and overrides genotoxin-induced G1 arrest [41]. These EBV-infected primary B cells are in type III latency and the suppression of p21^cip/waf1 ^is due to post-transcriptional regulation, whereas HL is in type II latency and the suppression of p21^cip/waf1 ^is regulated at the transcriptional level. Despite of the differences, the suppression of p21^cip/waf1 ^appears to be a common event critical for the development of these lymphomas.

At greater than 5*10^6 ^copies per cell, *EBERs *are the most abundant RNAs in EBV-infected cells. Although *EBERs *seem to prevent apoptosis through interacting with PKR, the nuclear localization of *EBERs *and cytoplasmic localization of PKR make a direct interaction unlikely [10]. Recently, *EBERs *were found to increase transcription or mRNA stability of IL-10, IL-9, or IGF1 in lymphoma or carcinoma cell lines [42,43]. With recognition of transcriptional regulation by *EBERs *and other noncoding RNAs as important biological processes [44], suppression of *p21*^cip/waf1 ^transcription deserves further investigations, because of the direct link with apoptosis and the known example of artificial microRNAs in regulating *p21*^cip/waf1 ^transcription [45].

Diepstra et al [46] reported a series of 412 HL patients with a median age of 35 years. In patients older than 50 years, the five-year failure free survival was 60% in EBV^+ ^cases *vs*. 85% in EBV^- ^cases (*p *= 0.01). Our series of 94 cases had a median age of 31 years. In patients older than 45 years, the 5-year disease free survival was 37% in EBV^+ ^cases *vs*. 74% in EBV^- ^cases (*p *= 0.02). In patients younger than 45 years, the 5-year disease free survival was 50% in EBV^+ ^cases *vs*. 67% in EBV^- ^cases (*p *= 0.17). Our data and those reported by Diepstra et al were consistent in showing that EBV^+ ^HL had a worse prognosis in the older age group.

In conclusion, *EBER1 *suppresses *p21*^cip1/waf1 ^transcription and confers resistance to drug-induced apoptosis in HL cell lines. Biologically, this anti-apoptotic activity might be important in the rescue of Reed-Sternberg cells and in the maintenance of the latent phase. Clinically, the suppression of p21^cip1/waf1 ^in EBV^+ ^HL predicts a worse prognosis, and the possibility of increased resistance to drug-induced apoptosis might have therapeutic implications.

## Conclusion

The anti-apoptotic activity of *EBER1 *is well known. In this study, we showed that *EBER1 *suppressed p21^cip1/waf1 ^in HL cell lines through down-regulation of p53, EGR1, and STAT1, and *EBER1*^+ ^HL cell lines were more resistant to apoptosis induced by histone deacetylase inhibitors or proteasome inhibitors. Because these drugs were known to act by increasing p21^cip1/waf1^, the anti-apoptotic activity of *EBER1 *was probably through the suppression of p21^cip1/waf1^. Clinically, EBV^+ ^HLs had weaker expression of p21^cip1/waf1 ^and a worse prognosis, which also supported a critical role of *EBER1 *in the rescue of Reed-Sternberg cells from apoptosis and in the clinical behaviors of HLs.

## Competing interests

The authors declare that they have no competing interests.

## Authors' contributions

TYL performed the most of experiments and wrote the manuscript. SJW analyzed the clinical data. MSH and FYL constructed the plasmids and the cell lines. MHT performed the bioinformatics on the microarray data. CHT established EBV-infected KMH2 and lymphoblastoid cell lines. SMH participated in drafting the manuscript. CWL coordinated the whole project and revised the manuscript. All authors read and approved the final manuscript.
